# What evidence exists of crop plants response to exposure to static magnetic and electromagnetic fields? A systematic map protocol

**DOI:** 10.1186/s13750-022-00292-w

**Published:** 2022-12-06

**Authors:** Agnieszka Pawełek, Samuel Acheaw Owusu, Daniele Cecchetti, Adrianna Zielińska, Joanna Wyszkowska

**Affiliations:** 1 Department of Plant Physiology and Biotechnology, Institute of Biology, Faculty of Biological and Veterinary Sciences, Nicolaus Copernicus University, Lwowska Street 1, 87-100 Toruń, Poland; 2 Faculty of Philosophy and Social Sciences, Nicolaus Copernicus University, Lwowska Street 1, 87-100 Toruń, Poland; 3 Department of Animal Physiology and Neurobiology, Institute of Biology, Faculty of Biological and Veterinary Sciences, Nicolaus Copernicus University, Lwowska Street 1, 87-100 Toruń, Poland

**Keywords:** Magnetic field, Electromagnetic field, Non-ionizing, Crops, Model plant, Mechanism of action, Evidence map, Growth and development

## Abstract

**Background:**

Increasing demand for food and concerns over the environmental impact of agriculture has prompted the search for alternatives to many conventional farming practices. Reports on exposing seeds and plants at various developmental stages to static magnetic field (SMF) and non-ionizing electromagnetic fields (EMF) as a form of priming indicate some positive effects on seed germinability, growth rate, resistance to stress conditions, and improved yield. However, there exist some inconsistent reported treatment protocols and contradictory study outcomes that make it difficult to draw objective conclusions on the potential use of SMF and EMF as sustainable alternatives to improving crop growth and yield. It is equally essential to understand any adverse effects of exposing plants to SMF and EMF considering the abundance of their sources in the environment. In order to provide a more coherent overview of how plants respond to exposure to SMF and EMF not only in their observed effects of agronomic importance but also in the mechanisms of action of SMF and EMF in plant cells, we prepare a systematic map.

**Methods:**

Literature will be identified by searching six bibliographic databases and three web-based search engines using terms obtained from the population, exposure, and outcome parameters of the research question. Primary research published in peer-reviewed journals and grey literature will be the source for the evidence map. Studies eligible for inclusion may involve: food crops and related research model plants exposed to SMF or non-ionizing EMF; treatment at all plant developmental stages excluding post-harvest improvement of food crops; and the presence of control groups. Eligible literature will be screened at the title, abstract, and full text levels. The validity of studies will not be critically appraised for the evidence map. A process of double extraction and coding of relevant information from eligible literature will be conducted. Within the evidence map, relevant data will be presented in the forms of text, graphs, tables, and figures. This will illustrate research trends, bring clarity to the evidence base concerning clusters of sufficient findings and areas of significant gaps, and inform stakeholders in decisions concerning research planning and policy formulation.

**Supplementary Information:**

The online version contains supplementary material available at 10.1186/s13750-022-00292-w.

## Background

The total demand for food worldwide between 2010 and 2050 has been projected to rise by 35% to 56% [1]. This projected increase in the demand for food products is manifested in both their quantity and quality. Producing enough food crops require that farming systems adapt to the changing climate while limiting their negative environmental impact. These concerns have prompted the search for alternative means to increase crop yield that reduce the use of farming inputs with high adverse effect on the ecosystem. These efforts to improve food crop production may be either newly discovered techniques or existing ones which have been enhanced in order to mitigate against unfavorable factors and maximize the potential yield of food crops.

Stakeholders currently rely on innovative plant breeding techniques [2] based often on genetic engineering to manage crop pests, diseases, and other stress conditions. However, other more conventional means of improving crop yield that have been utilized over time include the priming of seeds and other planting materials. These priming techniques, consisting of the application of water, salt solution, nanoparticles, osmotic solutions, essential inoculating microorganisms, or varying temperatures, have been used to improve germination, ensure uniform emergence, improve seedling growth rate, reduce susceptibility to diseases, improve resistance to drought, and overall increase crop yield [3]. Among priming methods, the use of physical techniques is considered as having lesser adverse environmental impact since they affect plants mainly through interference with physiological and biochemical processes occurring in different plant tissues [4]. This has led to increasing research interest in the effect of exposing plants to magnetic fields which may be present in the form of static magnetic field (SMF) or electromagnetic field (EMF). The biological processes in different plant species are reported to be influenced by SMFs [5, 6] resulting in changes in seed germinability [7, 8], root growth and development [9], seedling vigor [10], plant yield [11], and resistance to stress factors [12–15]. Similarly, exposure to EMFs is reported to affect the physiological parameters of plants involving growth and development [16–19] and actions at the molecular level such as gene expression and regulation [20–22].

Despite these potential applications of SMF and EMF to improve crop production, the exact mechanism of their actions in plant cells is poorly understood. Currently, a few studies have attempted to explain how SMF and EMF act in plant cells. It is postulated that Reactive Oxygen Species (ROS) and cytosolic calcium [23] as well as cryptochromes (blue-light receptors) and auxin signaling [9] are involved in plant growth regulated by magnetic fields. Gaining a better insight into how these magnetic fields act on plants could contribute to finding sustainable means of improving food crop production. Magnetic fields have natural and man-made sources commonly found in the environment [23]. Due to their proliferation, it is not enough to investigate their potential use as priming agents for improved crop production but also equally important to determine if and how they may adversely affect plant processes that influence their germinability, growth rate, response to stress factors, and yield.

There are many variations in the outcome of studies on the effect of plant exposure to SMFs and EMFs which may be attributed to differences in the treated plant species, the stage of plant development during treatment, experimental models used, and the treatment conditions. Concerns have been raised in some literature [24] about problems with the setup and description of treatment conditions including limited information on the experimental protocols in some studies. Although many of the studies investigating exposure effects of SMFs and EMFs on plants focus on traits of agronomic importance, it is essential to present an overview of relevant studies with outcomes of either positive, negative, or no significant effects. This evidence map will contribute to providing plant and environmental scientists, industries, and policymakers a reliable scope of the body of evidence to inform decisions on future research, investments in new crop production methods, and the formulation of policies by highlighting not only consensus and common traits within the studied plant species, treatment protocols, study designs, and outcomes but also points of contradictions and inconsistencies within these parameters. This study will differ from other secondary studies in a number of ways:

- It will cover a wider range of physical agents (SMFs and non-ionizing EMFs), thus, providing a more comprehensive view of the evidence base;
- It will bring more clarity to the potential use of SMFs and EMFs as priming agents for the sustainable production of food crops to meet a growing world population, thereby combining the interests of plants scientists, environmental scientists, industries, regulators, and policymakers;
- It aims to gather data on treatment outcomes whether positive, negative, or of no significant effects;
- It will include reported treatment or exposure parameters of all eligible studies to aid in the proper assessment and comparison of the different studies.

## Definitions: static magnetic field and electromagnetic fields

In our study, SMFs refer to constant fields, which do not change in intensity or direction over time. It can be created by magnets, by the flow of direct current (DC) electricity, or from natural sources. The earth generates its own magnetic field known as the geomagnetic field with a magnitude range between 30 and 70 µT [25]. A magnetic field can be represented as a vector and may be specified in one of two ways: as magnetic flux density, **B** or as magnetic field strength, **H**. The units of **B** and **H** are expressed in tesla (T) and amperes per meter (Am^−1^), respectively. In a vacuum and with good approximation in air, **B** and **H** are related through the magnetic permeability by the expression: **B** = *µH* (*µ* = 4π∙10^−7^H/m in a vacuum, air, and in biological materials).

Time-varying magnetic fields are produced by devices using alternating current (AC). They reverse their direction at a regular frequency (expressed in units of hertz, Hz) and can induce an electric current in a conductor present in this field. Exposure to time-varying magnetic fields results in internal body currents and energy absorption in tissues that depend on the coupling mechanisms and the frequency involved. Thus, the dosimetric quantities, taking into account different frequency, need to be specified: Current density, **J** (SI unit, Am^−2^) up to 10 MHz frequency range; Current, ***I*** (A), up to 110 MHz; Specific energy absorption rate, SAR (Wkg^−1^), 100 kHz–10 GHz range; Specific energy absorption, SA (Jkg^−1^), 300 MHz–10 GHz range; and Power density, **S** (Wm^−2^), 10 GHz–300 GHz range [26].

**Fig. 1:**
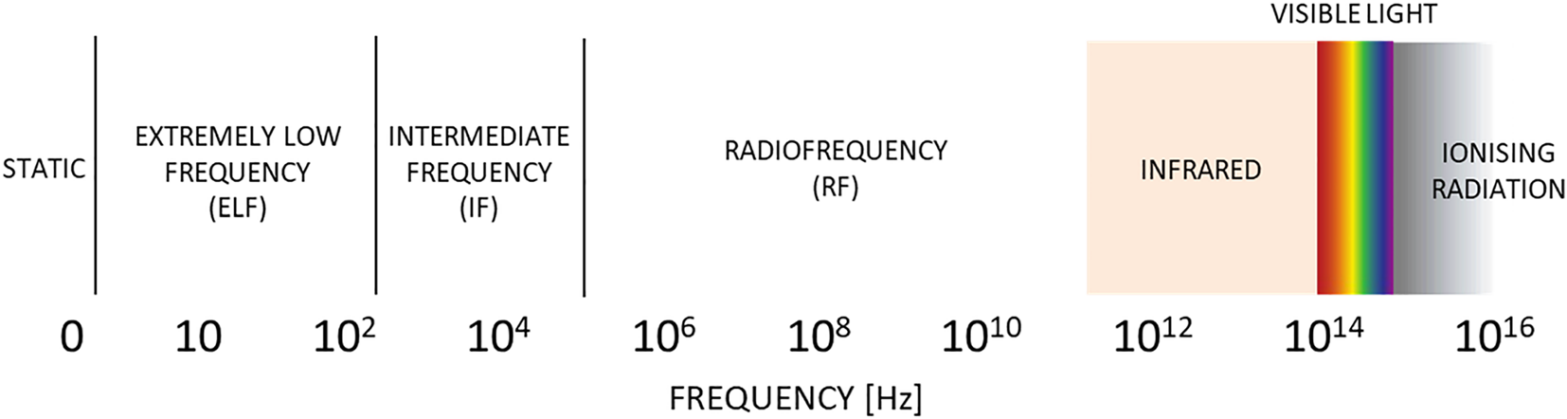
Illustration of the various frequency ranges of static magnetic field, non-ionizing electromagnetic field, and ionizing radiation

The mutual interaction of electric and magnetic fields (electric fields are produced by changing magnetic fields and circulating magnetic fields are produced by changing electric fields and by electric currents) produces EMFs. Therefore, in our study, we will use the concept of EMF to describe an alternating magnetic field. On the electromagnetic spectrum, there are different ranges of frequencies (Fig. 1) which are classified into non-ionizing EMFs and ionizing radiation. In contrast to ionizing radiation, the non-ionizing EMFs do not possess enough energy to remove electrons from atoms or molecules, thereby making them relatively safer. For this reason and the ubiquitous nature of the sources of non-ionizing EMF in our environment, the scope of this map will be limited to studies on the effects of exposure of food crops and related research model plants to SMF and non-ionizing EMF.

## Stakeholders’ involvement

The review question was formulated by the leading researchers in the review team who through their existing research activities have firsthand experience of the potential use of MFs as agents to influence the growth and development of food crops. The scope of the review in terms of the population group (food crops and related research model plants), exposure parameters related to SMF and non- ionizing EMF, and the eligibility criteria for included literature were determined by the entire research team based on gaps identified in existing primary and secondary studies. The research team through the membership of one of the leading reviewers to the Maria Skłodowska-Curie Polish Radiation Research Society and the Polish Society of Applied Electromagnetism, has access to external experts for consultation on the application of MFs should the need arise during the study.

## Objectives of the systematic map

Four main objectives have been set for mapping the existing knowledge base of food crop plants response to exposure to SMFs and EMFs:

1. Provide an overview of observed effects (positive or negative) of exposure to SMFs and EMFs in studied plants, particularly food crops;
2. Present the common traits in studied plant species, treatment protocols, and study outcomes (positive, negative, or no effect);
3. Identify knowledge gaps, contradictions, and inconsistencies in reported studies related to study designs, treated plant species, treatment protocols, and outcomes;
4. Assess the potential for further research on this and related topics.

## Primary research question

*What evidence exists of crop plants response to exposure to static magnetic and electromagnetic fields?*

## Elements of the primary research question

***Population***: food crops; related model plants.

***Exposure***: SMFs; any of the non-ionizing EMFs.

***Comparator***: treated and untreated groups of same plant species; treatment of different plant species; treatment at different stages of plant development or life cycles (pre-sowing, pre-emergence, or during growth); treatment of different propagating materials (seeds, pollen, or cuttings); exposure to different physical factors (SMF, radiofrequency, and other non-ionizing EMF); different exposure parameters including frequency (range), intensity, polarization, duration, and direction.

***Outcome***: positive, negative, or no changes of agronomic or environmental importance; effects on biological processes of plants related to germination, seedling emergence, growth, yield, and stress response; and the mechanisms of action (biophysical, cellular, biochemical, or molecular) of SMF and EMF in food crop plants.

## Secondary research questions

In order to fully address the primary research question, a number of secondary questions have been proposed:

- *What are some common traits of studied plants including species, treated planting materials (seeds, pollen, or cuttings) and their developmental stage at treatment (pre-sowing, pre-emergence, or during growth)?*
- *What are the commonly used protocols of treatment (including intensity and frequency, duration, or intermittent or continuous exposure)?*
- *What are the observed exposure effects on the physiological, cellular, biochemical, and molecular processes in plants?*
- *How is the available evidence characterized in terms of the type of study, study location (country), study setting (field/laboratory/greenhouse), plant species, plant organs treated or measured, control parameters, and bibliographic characteristics such as publication type?*
- *Are there any effects of potential agronomic, environmental, or economic interests?*
- *What are the potentials of SMFs and EMFs as priming agents for improved and sustainable food crop production?*
- *What are the areas of inconsistencies or contentions in reported outcomes?*
- *Which research areas are well studied with adequate consensus and which areas are less studied?*

## Methods

The systematic map will conform to the Collaboration for Environmental Evidence Guidelines and Standards for Evidence Synthesis in Environmental Management [27]. This protocol adheres to the RepOrting standards for Systematic Evidence Syntheses (ROSES) for systematic map protocols [28] which has been included as an additional file [see Additional file 1].

### Search terms and language

A number of English search terms were created from the population, exposure, and outcome elements of the research question. These population terms were determined by identifying common food crops and other parameters of studied plants through Google search, discussions among the study team members, and literature search in the Web of Science Core Collection (WOS CC) database. The exposure and outcome terms were determined by analyzing key articles and reports obtained through the WOS CC database and discussions within the study team. No unique terms related to the comparator elements were prepared.

The population search terms include general references to plants such as flora, crop, seeds, roots, leaves, pollen, and flowers. Additionally, terms related to crop groups such as cereal, vegetable, and grains, as well as the common and Latin names of food crops were included in the population terms. The exposure terms include references to magnetic fields, SMFs, non-ionizing EMFs, specific frequencies of the non-ionizing electromagnetic spectrum, and their commonly accepted abbreviations. A number of outcome terms commonly found in published studies were selected and include references to some plant physiological, cellular, metabolic, and molecular parameters such as germination, photosynthesis, plant growth, plant vigor, crop yield, phytohormones, and plant gene expression.

Subsequently, some search terms related to the application of magnetic fields in animal studies and in the medical sciences were identified by searching the WOS CC database. These search terms were used to improve the precision of the search strategy. More details of the search terms are presented in an additional file [see Additional file 2]. The individual search terms and how they were used to create the search strings are described in the next section.

### Search string formation and pilot literature search

The Boolean operator “OR” was used to string together the individual terms within each group of search terms. The population terms were subsequently combined with the outcome terms by the “OR” Boolean operator. It is reported that including outcome-related terms may limit the amount of literature retrieved because outcome is the least effective of the four PECO (PICO) elements to retrieve literature [29]. We avoided this limitation by combining the selected outcome terms and the population terms by using the “OR” Boolean operator, thereby expanding the possible literature to be retrieved. The results were subsequently combined with the exposure terms by using the “AND” Boolean operator. These searches were done in the Title field of the WOS CC database. Searching in the Title field was sensitive enough to effectively retrieve relevant literature due to the numerous population, outcome, and exposure terms used in the search strategy. We improved on the balance between the search sensitivity and precision by eliminating from the search results, some literature related to animals and the medical sciences by using the “NOT” Boolean operator. These searches were done in the Title, Author Keywords, and Keywords Plus fields of the WOS CC database. We utilized the (*) wildcard to expand some search terms for a more comprehensive search result.

*Population terms* (cereal OR vegetable OR grains OR Sorghum OR “guinea corn” OR millet OR fonio OR teff OR rice OR wheat OR buckwheat OR amaranth OR quinoa OR maize OR corn OR rye OR oat OR barley OR triticale OR lentil OR okra OR zucchini OR squash OR gourd OR pumpkin OR tomato OR pea OR chickpea OR cabbage OR broccoli OR cauliflower OR kale OR lettuce OR onion OR shallot OR garlic OR radish OR spinach OR horseradish OR groundnut OR peanut OR pepper OR carrot OR celery OR cucumber OR eggplant OR melon OR watermelon OR cowpea OR bean OR soybean OR potato OR sunflower OR canola OR rapeseed OR beetroot OR turnip OR coffee OR fig OR tea OR “Sorghum bicolor” OR “Sorghum vulgare” OR “Setaria italica” OR “Eleusine coracana” OR “Digitaria exilis” OR “Echinochloa frumentacea” OR “Echinochloa esculenta” OR “Panicum miliaceum” “Pennisetum glaucum” OR “Digitaria sanguinalis” OR “Eragrostis tef” OR “Oryza sativa” OR “Triticum aestivum” OR “Triticum durum” OR “Triticum spelta” OR “Triticum monococcum” OR “Triticum turanicum” OR “Fagopyrum esculentum” OR “Amaranthus cruentus” OR “Chenopodium quinoa” OR “Zea mays” OR “Secale cereale” OR “Avena sativa” OR “Hordeum vulgare” OR “x Triticosecale” OR “Lens culinaris” OR “Lens esculenta” OR “Abelmoschus esculentus” OR “Abelmoschus caillei” OR “Cucurbita pepo” OR “Cucurbita moschata” OR “Cucurbita mixta” OR “Cucurbita maxima” OR “Benincasa hispida” OR “Solanum lycopersicum” OR “Cajanus cajan” OR “Cicer arietinum” OR Brassica OR “Brassica oleracea var. capitata” OR “Brassica oleracea var. italica” OR “Brassica oleracea var. botrytis” OR “Brassica oleracea var. acephala” OR “Lactuca sativa” OR “Allium cepa” OR “Allium sativum” OR “Raphanus sativus” OR “Spinacia oleracea” OR “Armoracia rusticana” OR “Cochlearia armoracia” OR Capsicum OR “Capsicum annuum” OR “Daucus carota” OR “Apium graveolens” OR “Cucumis sativus” OR “Solanum melongena” OR “Solanum aethiopicum” OR “Cucumis melo” OR “Citrullus lanatus” OR “Citrullus vulgaris” OR “Vigna unguiculata” OR “Vigna sinensis” OR “Vicia faba” OR “Vigna radiata” OR Phaseolus OR “Phaseolus vulgaris” OR “Phaseolus aureus” OR “Phaseolus radiatus” OR “Glycine max” OR “Helianthus annuus” OR “Brassica napus” OR “Beta vulgaris” OR “Brassica rapa var. rapa” OR “Coffea arabica” OR “Ficus carica” OR Camellia sinensis OR Arabidopsis OR “Arabidopsis thaliana” OR tobacco OR “Nicotiana tabacum” OR “Nicotiana benthamiana” OR Medicago OR “Medicago truncatula” OR root* OR stem* OR pollen OR flower* OR seed* OR leaf OR leaves OR cuttings OR (Pre-sow* AND (exposure or treatment)) or Flora OR crop* OR plant*).

OR

*Outcome terms* (germinat* OR germinability OR (growth AND (crop OR crops OR plant OR plants)) OR photosynthe* OR flower* OR yield* OR “gene expression” OR gene-expression OR “gene regulation” OR “mechanism of action” OR vigor OR vigour OR chlorophyll OR phytohormone* OR cryptochrome OR “reactive oxygen species” OR “ROS”).

AND

*Exposure terms* ((magnetic AND (static OR field* OR priming OR treatment* OR effect or exposure)) OR magnetic-field OR “MF” OR “SMF” OR magnetoprim* OR magneto-prim* OR “biomagnetic priming” OR magnetoreception OR magneto-reception OR magnetoperception OR magneto-perception OR (electromagnetic AND (puls* OR field* OR spectrum OR priming OR treatment* OR effect or exposure)) OR “EMF” OR non-ionizing OR non-ionising OR nonioniz* OR nonionis* OR “non ionizing” OR “non ionising” OR non-ionized OR non-ionised OR “radio frequency” OR “radio frequencies” OR “RF” OR radiofrequency OR radiofrequencies OR radio-frequency OR radio-frequencies OR “extremely low frequency” OR “extremely low frequencies” OR “ELF” OR “very Low Frequency” OR “very Low Frequencies” OR “VLF” OR “low frequency” OR “low frequencies” OR “LF” OR “medium frequency” OR “medium frequencies” OR “MF” OR “high frequency” OR “high frequencies” OR “very high frequency” OR “very high frequencies” OR “VHF” OR “ultra high frequency” OR “ultra high frequencies” OR “UHF” OR “super high frequency” OR “super high frequencies” OR “extremely high frequency” OR “extremely high frequencies” OR “EHF” OR “GSM” OR “2G” OR “3G” OR “4G” OR “5G” OR infrared OR “IR”).

NOT

*Terms related to animals and medical science* (human OR animal OR animals OR mammal* OR mice OR mouse OR cancer OR leukemia OR leukaemia OR “stem cell” OR “stem cells” OR bone OR child* OR patient* OR brain OR transcranial OR “magnetic resonance” OR surgery OR clinical OR ablation OR medicine OR rat OR rats OR pig OR pigs).

This search strategy piloted in the WOS CC database can be found together with the description of the search terms in an additional Microsoft Word file [see Additional file 2].

### Databases for literature search

The literature search process as piloted in the WOS CC database will be adapted for use in the following databases for which the researchers have access to through their institutions: Scopus; Agricola (Ebscohost Platform); GreenFile (EBSCOhost Platform); Agris; and OpenDissertations (Ebscohost Platform).

Additionally, three web-based search engines—Bielefeld Academic Search Engine (BASE), EMF Portal, and Google Scholar—will be searched for more literature which may not have been retrieved through the previously listed databases. The search results from Google Scholar will be sorted according to relevance and the first 100 results will be screened for eligible literature. For every literature search conducted in each database, search-engine, or journal, records will be made of the date of search, number of hits, name of platform, and the names of reviewers performing the literature search. Where necessary, authors of published articles and reviews will be contacted for recommendations for grey literature. During the search process, no limitations based on the geographical location of the study or the type of literature (primary research articles or secondary studies) will be applied.

### Estimating the comprehensiveness of the search

We have compiled a list of 24 benchmark articles we consider to be relevant to the research question. These articles report on studies involving exposure of different plant species to either SMF or non-ionizing EMF and meet the various criteria for inclusion in the systematic map. In order to examine the comprehensiveness of the search strategy, we tested its ability to retrieve these benchmark articles in our pilot search in WOS CC database. All the articles were successfully retrieved by the search strategy [see Additional file 3]. In subsequent literature searches, the search strategy will be adapted for each database and search engine.

## Article screening and study eligibility criteria

### Screening process for relevant literature

Articles retrieved after the search process will be assessed for inclusion in the systematic map based on a pre-defined set of eligibility criteria. This screening process will be divided among the reviewers and in cases where a reviewer has authored any of the retrieved articles, such articles will be assigned to other reviewers for screening.

The articles retrieved from the search process will be gathered in the Zotero reference management tool. Duplicate articles will thereafter be eliminated. To assess the effectiveness and transparency of the de-duplication process, it will be repeated in the Mendeley reference management tool. Articles retrieved will first be screened by their titles to eliminate those not relevant to the study. Abstracts of articles that pass this initial screening process will be further assessed for eligibility for inclusion. The full text of all literature that pass the abstract screening stage will then be screened.

### Test for consistency in screening

Two hundred set of articles will be randomly selected and screened independently by all the reviewers at both the title and abstract screening phases. The results of these independent screening will be assessed and any disagreements resolved until at least a kappa score of 0.6 is obtained. This will ensure the reviewers have an agreement to an acceptable level of what qualifies an article for inclusion in the map.

This process of testing for consistency will be repeated at the full text screening phase where among the articles selected for screening, 50 will be randomly selected for consistency test among the reviewers. At this stage, any inconsistency in screening among the reviewers will be discussed, the eligibility criteria will be clarified, and all disagreements will be resolved.

### Eligibility criteria

The criteria by which eligible literature will be selected at the title, abstract, and full text phases as described previously have been divided into the population, exposure, comparator, outcome and study type elements of the research question. We have also defined other bibliographic criteria for eligibility. At the full text screening phase, we will make records of all literature that do not meet the eligibility criteria in an additional file and the reasons for their exclusion will be noted.

#### Relevant population

Only studies that include food crops and related model plants for laboratory research will be included in this evidence map. Studies involving only animals, humans, plants grown solely for ornamental or medicinal purposes, other non-food crops, yeast, or algae among others will be excluded from this evidence map. There will be no limitations based on the type of plant cultivation material used in the study or the stage of development of the plant during the study.

#### Relevant exposure

Only studies involving exposure of relevant plant species to SMFs or non-ionizing EMFs will be eligible for inclusion in the evidence map. Studies involving only treatment with ionizing electromagnetic radiation will be ineligible for inclusion. Eligible articles must present adequate information on the exposure parameters including intensity, frequency, period, duration, and method of exposure. Exposure may occur before cultivation (pre-sowing), before seedling emergence from soil, or during growth of the seedling or mature plant. All post-harvest treatments meant to improve the yield output and preservation of the crop will be excluded.

#### Relevant comparators

Articles eligible for inclusion must indicate the presence of a comparator group of plant species or treatment protocol. Comparator groups may include: control plants (no exposure); same plant species under different exposure parameters; similar exposure parameters on different plant species; different location of study (e.g., laboratory test vs field test); or different growth media. Articles that report no comparator groups will be excluded.

#### Relevant outcomes

For eligible study outcomes, exposure to SMF or EMF may either cause positive or negative significant effects or no significant effect at all on the studied traits in plants. Observed outcomes may concern physiological, cellular, biochemical, or molecular effects. Eligible literature may include reports on the mechanism of action of SMF and EMF in plants. Other relevant outcomes include exposure effect on growth, yield, and stress resistance parameters, among others.

#### Relevant study type

Primary experimental or quasi-experimental studies with control groups will be eligible for inclusion. Secondary studies including reviews and meta-analysis and theoretical works will be excluded. Literature on primary studies regardless of scale or setting (laboratory, greenhouse, or field) will be selected if they meet the other defined eligibility requirements.

#### Other bibliographic criteria

***Type of publication: ***Only peer-reviewed articles and grey literature including unpublished manuscripts, dissertations, and institutional reports which are based on primary research data will be selected for inclusion in the systematic map. Books, news items, editorials, opinion pieces, and reports not based on primary studies will be ineligible for inclusion. The reference list of appropriate books, book chapters, reviews and meta-analysis will be assessed for relevant literature not retrieved during the literature search.

***Date of publication***: No limitations will be placed on the date of publication.

***Language:*** Only articles published in English and Polish will be selected due to limited resources to translate other languages.

***Full text availability:*** Studies whose full text is available to the review team will be included. Attempt will be made to contact authors of studies that pass the title and abstract screening phase if their full text is unavailable to the review team.

### Critical appraisal of studies

The main objective of the systematic map is to present a broad overview of existing evidence of the effect of exposing food crop plants to SMFs or non-ionizing EMFs and the current progress being made in this field. Thus, no critical appraisal of the studies included in this map will be done. However, basic records will be made of the study setting and description of the study design.

## Data extraction and coding strategy

Reviewers will be assigned a set number of literature that passes the final full text screening to extract data. This work will then be cross-checked by a second reviewer. Any differences between the two reviewers will be discussed and consensus sought. Among the categories of data to be extracted and coded are the following:

- Names of reviewers;
- Bibliographic information including title, author(s), abstract, keywords, journal, and geographic location of the study;
- Population parameters including the names and classifications of the studied plants and the plant developmental stage during treatment;
- Exposure parameters including type of treatment source, intensity, duration of exposure, and the method and consistency of exposure;
- Comparator parameters including differences in the control and treated plant species and their exposure (treatment) conditions;
- Study outcome parameters including SMF and non-ionizing EMF mechanism of action in plants and their effect on plant growth, yield, and response to stress;
- Study design parameters such as type of study, context of study, sensitivity of the measuring devices, and climate and other growth conditions.

A data extraction guide containing details of the data extraction strategy has been designed and is available as an additional file [see Additional file 4]. This guide is inspired by a sample form from another systematic map protocol [30].

## Data synthesis and presentation

Data extracted from studies that meet the eligibility criteria will be presented in a published systematic map report. The entire review process will be described in the report. Relevant data that answers the review questions will be illustrated in text, graphical forms, tables, and figures. The report will include information on studied plant species, treatment protocols, particularly on dose, and reported outcomes. Areas of consensus on study outcomes, limitations within the existing knowledge base, and their implications for future research and policy formulation will be highlighted. The reviewers will compare the clusters of sufficient knowledge base with those containing substantial gaps on a heat map. This will help determine the possibility for further studies including primary research and systematic reviews. Information presented in the systematic map will include the following:

- Major plants and their characteristics studied for their response to exposure to SMF and non-ionizing EMF;
- Treatment source and their exposure parameters;
- Geographical representation and concentration of studies;
- Areas of consensus and inconsistencies in treatment setup and study outcomes;
- Clusters of sufficient and insufficient knowledge base to inform further research;
- Trends in publication of studies.

## Supplementary Information

**Additional file 1.** ROSES form for systematic map protocol.

**Additional file 2.** Search terms and search strategy.

**Additional file 3.** Benchmark articles and test of search comprehensiveness.

**Additional file 4.** Data extraction guide.

## Data Availability

Data sharing is not applicable to this protocol as no datasets were generated or analyzed in preparing this protocol.
