# Supplementary material for: What evidence exists of crop plants response to exposure to static magnetic and electromagnetic fields? A systematic map protocol
Source: Environ Evid. 2022 Dec 6;11:37. doi: 10.1186/s13750-022-00292-w (PMC11378831; doi:10.1186/s13750-022-00292-w)
Supplement: Supplementary file 2 — Additional file 2. Search terms and search strategy. [file 13750_2022_292_MOESM2_ESM.docx]

**Search terms and their description**

| **Element of research question** | **Description** | **Group of search terms piloted in Web of Science** |
| --- | --- | --- |
| Population search terms | Common crop groups | Cereal OR vegetable OR grains |
|  | Common names of crops | Sorghum OR “guinea corn” OR Millet OR fonio OR Teff OR Rice OR Wheat OR Buckwheat OR amaranth OR quinoa OR Maize OR corn OR rye OR oat OR barley OR triticale OR lentil OR okra OR Zucchini OR squash OR gourd OR Pumpkin OR Tomato OR Pea OR chickpea OR Cabbage OR Broccoli OR Cauliflower OR Kale OR Lettuce OR Onion OR shallot OR garlic OR Radish OR Spinach OR Horseradish OR groundnut OR peanut OR Pepper OR Carrot OR Celery OR Cucumber OR Eggplant OR Melon OR watermelon OR Cowpea OR Bean OR Soybean OR potato OR Sunflower OR Canola OR rapeseed OR Beetroot OR Turnip OR coffee OR fig OR tea |
|  | Latin names of crops | “Sorghum bicolor” OR “Sorghum vulgare” OR “Setaria italica” OR “Eleusine coracana” OR “Digitaria exilis” OR “Echinochloa frumentacea” OR “Echinochloa esculenta” OR “Panicum miliaceum” “Pennisetum glaucum” OR “Digitaria sanguinalis” OR “Eragrostis tef” OR “Oryza sativa” OR “Triticum aestivum” OR “Triticum durum” OR “Triticum spelta” OR “Triticum monococcum” OR “Triticum turanicum” OR “Fagopyrum esculentum” OR “Amaranthus cruentus” OR “Chenopodium quinoa” OR “Zea mays” OR “Secale cereale” OR “Avena sativa” OR “Hordeum vulgare” OR “x Triticosecale” OR “Lens culinaris” OR “Lens esculenta” OR “Abelmoschus esculentus” OR “Abelmoschus caillei” OR “Cucurbita pepo” OR “Cucurbita moschata” OR “Cucurbita mixta” OR “Cucurbita maxima” OR “Benincasa hispida” OR “Solanum lycopersicum” OR “Cajanus cajan” OR “Cicer arietinum” OR Brassica OR “Brassica oleracea var. capitata” OR “Brassica oleracea var. italica” OR “Brassica oleracea var. botrytis” OR “Brassica oleracea var. acephala” OR “Lactuca sativa” OR “Allium cepa” OR “Allium sativum” OR “Raphanus sativus” OR “Spinacia oleracea” OR “Armoracia rusticana” OR “Cochlearia armoracia” OR Capsicum OR “Capsicum annuum” OR “Daucus carota” OR “Apium graveolens” OR “Cucumis sativus” OR “Solanum melongena” OR “Solanum aethiopicum” OR “Cucumis melo” OR “Citrullus lanatus” OR “Citrullus vulgaris” OR “Vigna unguiculata” OR “Vigna sinensis” OR “Vicia faba” OR “Vigna radiata” OR Phaseolus OR “Phaseolus vulgaris” OR “Phaseolus aureus” OR “Phaseolus radiatus” OR “Glycine max” OR “Helianthus annuus” OR “Brassica napus” OR “Beta vulgaris” OR “Brassica rapa var. rapa” OR “Coffea arabica” OR “Ficus carica” OR Camellia sinensis OR Arabidopsis OR “Arabidopsis thaliana” OR tobacco OR “Nicotiana tabacum” OR “Nicotiana benthamiana” OR Medicago OR “Medicago truncatula” |
|  | Plant organs or planting materials treated or observed for treatment effect | Root* OR stem* OR pollen OR flower* OR seed* OR leaf OR leaves OR cuttings |
|  | Period of crop cultivation for treatment.  *Note that other the search results may include studies where treatment occurred after sowing and these search terms would not eliminate those studies. | Pre-sow* AND (exposure or treatment) |
|  | General reference to plants and crops | Flora OR crop* OR plant* |
| Exposure search terms | Terms related to static magnetic fields and exposure to them | (Magnetic AND (static OR field* OR priming OR treatment* OR effect or exposure)) OR magnetic-field OR “MF” OR “SMF” OR Magnetoprim* OR magneto-prim* OR “biomagnetic priming” OR magnetoreception OR magneto-reception OR magnetoperception OR magneto-perception OR |
|  | Terms related to non-ionizing electromagnetic fields and exposure to them | (Electromagnetic AND (puls* OR field* OR spectrum OR priming OR treatment* OR effect or exposure)) OR “EMF” OR Non-ionizing OR non-ionising OR nonioniz* OR nonionis* OR “non ionizing” OR “non ionising” OR non-ionized OR non-ionised |
|  | Specific examples of non-ionizing electromagnetic fields | “Radio Frequency” OR “Radio Frequencies” OR “RF” OR radiofrequency OR radiofrequencies OR radio-frequency OR radio-frequencies OR “extremely low frequency” OR “extremely low frequencies” OR “ELF” OR “very Low Frequency” OR “very Low Frequencies” OR “VLF” OR “Low Frequency” OR “Low Frequencies” OR “LF” OR “Medium Frequency” OR “Medium Frequencies” OR “MF” OR “High Frequency” OR “High Frequencies” OR “Very High Frequency” OR “Very High Frequencies” OR “VHF” OR “Ultra High Frequency” OR “Ultra High Frequencies” OR “UHF” OR “Super High Frequency” OR “Super High Frequencies” OR “Extremely High Frequency” OR “Extremely High Frequencies” OR “EHF” OR “GSM” OR “2G” OR “3G” OR “4G” OR “5G” OR infrared OR “IR” |
| Outcome search terms | Reference to some plant physiological, molecular, metabolic, and cellular parameters. | Germinat* OR germinability OR (Growth AND (crop OR plant)) OR Photosynthe* OR Flower* OR yield* OR “Gene expression” OR gene-expression OR “gene regulation” OR “mechanism of action” OR vigor OR vigour OR chlorophyll OR phytohormone* OR cryptochrome OR “reactive oxygen species” OR “ROS” |
| Animal and medical science related terms | These are common terms appearing in titles and keywords of literature that are not within the scope of this study | Human OR animal OR mammal* OR mice OR mouse OR cancer OR leukemia OR leukaemia OR “stem cell” OR bone OR child* OR patient* OR brain OR transcranial OR “magnetic resonance” OR surgery OR clinical OR ablation OR medicine OR rat OR pig |

**SEARCH STRATEGY**

**Date: 18.09.2022**

**Database: Web of Science CC**

**Literature search performed by: AP, SOA, DC, AZ, & JW**

| **Step #** | **Field** | **Search** | **Hits** | **String** |
| --- | --- | --- | --- | --- |
| 12 |  | English AND Polish | 9,867 |  |
| 11 |  | #9 NOT #10 | 10,513 |  |
| 10 | Keyword Plus | Human OR animal OR animals OR mammal* OR mice OR mouse OR cancer OR leukemia OR leukaemia OR “stem cell” OR “stem cells” OR bone OR child* OR patient* OR brain OR transcranial OR “magnetic resonance” OR surgery OR clinical OR ablation OR medicine OR rat OR rats OR pig OR pigs | 5,610,780 | KP=(Human OR animal OR animals OR mammal* OR mice OR mouse OR cancer OR leukemia OR leukaemia OR “stem cell” OR “stem cells” OR bone OR child* OR patient* OR brain OR transcranial OR “magnetic resonance” OR surgery OR clinical OR ablation OR medicine OR rat OR rats OR pig OR pigs) |
| 9 |  | #7 NOT #8 | 10,785 |  |
| 8 | Author Keywords | Human OR animal OR animals OR mammal* OR mice OR mouse OR cancer OR leukemia OR leukaemia OR “stem cell” OR “stem cells” OR bone OR child* OR patient* OR brain OR transcranial OR “magnetic resonance” OR surgery OR clinical OR ablation OR medicine OR rat OR rats OR pig OR pigs | 3,129,955 | AK=(Human OR animal OR animals OR mammal* OR mice OR mouse OR cancer OR leukemia OR leukaemia OR “stem cell” OR “stem cells” OR bone OR child* OR patient* OR brain OR transcranial OR “magnetic resonance” OR surgery OR clinical OR ablation OR medicine OR rat OR rats OR pig OR pigs) |
| 7 |  | #5 NOT #6 | 10,913 |  |
| 6 | Title | Human OR animal OR animals OR mammal* OR mice OR mouse OR cancer OR leukemia OR leukaemia OR “stem cell” OR “stem cells” OR bone OR child* OR patient* OR brain OR transcranial OR “magnetic resonance” OR surgery OR clinical OR ablation OR medicine OR rat OR rats OR pig OR pigs | 11,255,725 | TI=(Human OR animal OR animals OR mammal* OR mice OR mouse OR cancer OR leukemia OR leukaemia OR “stem cell” OR “stem cells” OR bone OR child* OR patient* OR brain OR transcranial OR “magnetic resonance” OR surgery OR clinical OR ablation OR medicine OR rat OR rats OR pig OR pigs) |
| 5 |  | #3 AND #4 | 13,111 |  |
| 4 | Title | (magnetic AND (static OR field* OR priming OR treatment* OR effect or exposure)) OR magnetic-field OR “MF” OR “SMF” OR Magnetoprim* OR magneto-prim* OR “biomagnetic priming” OR magnetoreception OR magneto-reception OR magnetoperception OR magneto-perception OR (Electromagnetic AND (puls* OR field* OR spectrum OR priming OR treatment* OR effect or exposure)) OR “EMF” OR Non-ionizing OR non-ionising OR nonioniz* OR nonionis* OR “non ionizing” OR “non ionising” OR non-ionized OR non-ionised OR “Radio Frequency” OR “Radio Frequencies” OR “RF” OR radiofrequency OR radiofrequencies OR radio-frequency OR radio-frequencies OR “extremely low frequency” OR “extremely low frequencies” OR “ELF” OR “very Low Frequency” OR “very Low Frequencies” OR “VLF” OR “Low Frequency” OR “Low Frequencies” OR “LF” OR “Medium Frequency” OR “Medium Frequencies” OR “MF” OR “High Frequency” OR “High Frequencies” OR “Very High Frequency” OR “Very High Frequencies” OR “VHF” OR “Ultra High Frequency” OR “Ultra High Frequencies” OR “UHF” OR “Super High Frequency” OR “Super High Frequencies” OR “Extremely High Frequency” OR “Extremely High Frequencies” OR “EHF” OR “GSM” OR “2G” OR “3G” OR “4G” OR “5G” OR infrared OR “IR” | 683,172 | TI=((magnetic AND (static OR field* OR priming OR treatment* OR effect or exposure)) OR magnetic-field OR “MF” OR “SMF” OR Magnetoprim* OR magneto-prim* OR “biomagnetic priming” OR magnetoreception OR magneto-reception OR magnetoperception OR magneto-perception OR (Electromagnetic AND (puls* OR field* OR spectrum OR priming OR treatment* OR effect or exposure)) OR “EMF” OR Non-ionizing OR non-ionising OR nonioniz* OR nonionis* OR “non ionizing” OR “non ionising” OR non-ionized OR non-ionised OR “Radio Frequency” OR “Radio Frequencies” OR “RF” OR radiofrequency OR radiofrequencies OR radio-frequency OR radio-frequencies OR “extremely low frequency” OR “extremely low frequencies” OR “ELF” OR “very Low Frequency” OR “very Low Frequencies” OR “VLF” OR “Low Frequency” OR “Low Frequencies” OR “LF” OR “Medium Frequency” OR “Medium Frequencies” OR “MF” OR “High Frequency” OR “High Frequencies” OR “Very High Frequency” OR “Very High Frequencies” OR “VHF” OR “Ultra High Frequency” OR “Ultra High Frequencies” OR “UHF” OR “Super High Frequency” OR “Super High Frequencies” OR “Extremely High Frequency” OR “Extremely High Frequencies” OR “EHF” OR “GSM” OR “2G” OR “3G” OR “4G” OR “5G” OR infrared OR “IR”) |
| 3 |  | #1 OR #2 | 3,085,777 |  |
| 2 | Title | Germinat* OR germinability OR (Growth AND (crop OR crops OR plant OR plants)) OR Photosynthe* OR Flower* OR Yield* OR “Gene expression” OR gene-expression OR “gene regulation” OR “mechanism of action” OR vigor OR vigour OR chlorophyll OR phytohormone* OR cryptochrome OR “reactive oxygen species” OR “ROS” | 555,322 | TI=(Germinat* OR germinability OR (Growth AND (crop OR crops OR plant OR plants)) OR Photosynthe* OR Flower* OR Yield* OR “Gene expression” OR gene-expression OR “gene regulation” OR “mechanism of action” OR vigor OR vigour OR chlorophyll OR phytohormone* OR cryptochrome OR “reactive oxygen species” OR “ROS”) |
| 1 | Title | Cereal OR vegetable OR grains OR Sorghum OR “guinea corn” OR Millet OR fonio OR Teff OR Rice OR Wheat OR Buckwheat OR amaranth OR quinoa OR Maize OR corn OR rye OR oat OR barley OR triticale OR lentil OR okra OR Zucchini OR squash OR gourd OR Pumpkin OR Tomato OR Pea OR chickpea OR Cabbage OR Broccoli OR Cauliflower OR Kale OR Lettuce OR Onion OR shallot OR garlic OR Radish OR Spinach OR Horseradish OR groundnut OR peanut OR Pepper OR Carrot OR Celery OR Cucumber OR Eggplant OR Melon OR watermelon OR Cowpea OR Bean OR Soybean OR potato OR Sunflower OR Canola OR rapeseed OR Beetroot OR Turnip OR coffee OR fig OR tea OR “Sorghum bicolor” OR “Sorghum vulgare” OR “Setaria italica” OR “Eleusine coracana” OR “Digitaria exilis” OR “Echinochloa frumentacea” OR “Echinochloa esculenta” OR “Panicum miliaceum” “Pennisetum glaucum” OR “Digitaria sanguinalis” OR “Eragrostis tef” OR “Oryza sativa” OR “Triticum aestivum” OR “Triticum durum” OR “Triticum spelta” OR “Triticum monococcum” OR “Triticum turanicum” OR “Fagopyrum esculentum” OR “Amaranthus cruentus” OR “Chenopodium quinoa” OR “Zea mays” OR “Secale cereale” OR “Avena sativa” OR “Hordeum vulgare” OR “x Triticosecale” OR “Lens culinaris” OR “Lens esculenta” OR “Abelmoschus esculentus” OR “Abelmoschus caillei” OR “Cucurbita pepo” OR “Cucurbita moschata” OR “Cucurbita mixta” OR “Cucurbita maxima” OR “Benincasa hispida” OR “Solanum lycopersicum” OR “Cajanus cajan” OR “Cicer arietinum” OR Brassica OR “Brassica oleracea var. capitata” OR “Brassica oleracea var. italica” OR “Brassica oleracea var. botrytis” OR “Brassica oleracea var. acephala” OR “Lactuca sativa” OR “Allium cepa” OR “Allium sativum” OR “Raphanus sativus” OR “Spinacia oleracea” OR “Armoracia rusticana” OR “Cochlearia armoracia” OR Capsicum OR “Capsicum annuum” OR “Daucus carota” OR “Apium graveolens” OR “Cucumis sativus” OR “Solanum melongena” OR “Solanum aethiopicum” OR “Cucumis melo” OR “Citrullus lanatus” OR “Citrullus vulgaris” OR “Vigna unguiculata” OR “Vigna sinensis” OR “Vicia faba” OR “Vigna radiata” OR Phaseolus OR “Phaseolus vulgaris” OR “Phaseolus aureus” OR “Phaseolus radiatus” OR “Glycine max” OR “Helianthus annuus” OR “Brassica napus” OR “Beta vulgaris” OR “Brassica rapa var. rapa” OR “Coffea arabica” OR “Ficus carica” OR Camellia sinensis OR Arabidopsis OR “Arabidopsis thaliana” OR tobacco OR “Nicotiana tabacum” OR “Nicotiana benthamiana” OR Medicago OR “Medicago truncatula” OR Root* OR stem* OR pollen OR flower* OR seed* OR leaf OR leaves OR cuttings OR (Pre-sow* AND (exposure or treatment)) or Flora OR crop* OR plant* | 2,739,444 | TI=(Cereal OR vegetable OR grains OR Sorghum OR “guinea corn” OR Millet OR fonio OR Teff OR Rice OR Wheat OR Buckwheat OR amaranth OR quinoa OR Maize OR corn OR rye OR oat OR barley OR triticale OR lentil OR okra OR Zucchini OR squash OR gourd OR Pumpkin OR Tomato OR Pea OR chickpea OR Cabbage OR Broccoli OR Cauliflower OR Kale OR Lettuce OR Onion OR shallot OR garlic OR Radish OR Spinach OR Horseradish OR groundnut OR peanut OR Pepper OR Carrot OR Celery OR Cucumber OR Eggplant OR Melon OR watermelon OR Cowpea OR Bean OR Soybean OR potato OR Sunflower OR Canola OR rapeseed OR Beetroot OR Turnip OR coffee OR fig OR tea OR “Sorghum bicolor” OR “Sorghum vulgare” OR “Setaria italica” OR “Eleusine coracana” OR “Digitaria exilis” OR “Echinochloa frumentacea” OR “Echinochloa esculenta” OR “Panicum miliaceum” “Pennisetum glaucum” OR “Digitaria sanguinalis” OR “Eragrostis tef” OR “Oryza sativa” OR “Triticum aestivum” OR “Triticum durum” OR “Triticum spelta” OR “Triticum monococcum” OR “Triticum turanicum” OR “Fagopyrum esculentum” OR “Amaranthus cruentus” OR “Chenopodium quinoa” OR “Zea mays” OR “Secale cereale” OR “Avena sativa” OR “Hordeum vulgare” OR “x Triticosecale” OR “Lens culinaris” OR “Lens esculenta” OR “Abelmoschus esculentus” OR “Abelmoschus caillei” OR “Cucurbita pepo” OR “Cucurbita moschata” OR “Cucurbita mixta” OR “Cucurbita maxima” OR “Benincasa hispida” OR “Solanum lycopersicum” OR “Cajanus cajan” OR “Cicer arietinum” OR Brassica OR “Brassica oleracea var. capitata” OR “Brassica oleracea var. italica” OR “Brassica oleracea var. botrytis” OR “Brassica oleracea var. acephala” OR “Lactuca sativa” OR “Allium cepa” OR “Allium sativum” OR “Raphanus sativus” OR “Spinacia oleracea” OR “Armoracia rusticana” OR “Cochlearia armoracia” OR Capsicum OR “Capsicum annuum” OR “Daucus carota” OR “Apium graveolens” OR “Cucumis sativus” OR “Solanum melongena” OR “Solanum aethiopicum” OR “Cucumis melo” OR “Citrullus lanatus” OR “Citrullus vulgaris” OR “Vigna unguiculata” OR “Vigna sinensis” OR “Vicia faba” OR “Vigna radiata” OR Phaseolus OR “Phaseolus vulgaris” OR “Phaseolus aureus” OR “Phaseolus radiatus” OR “Glycine max” OR “Helianthus annuus” OR “Brassica napus” OR “Beta vulgaris” OR “Brassica rapa var. rapa” OR “Coffea arabica” OR “Ficus carica” OR Camellia sinensis OR Arabidopsis OR “Arabidopsis thaliana” OR tobacco OR “Nicotiana tabacum” OR “Nicotiana benthamiana” OR Medicago OR “Medicago truncatula” OR Root* OR stem* OR pollen OR flower* OR seed* OR leaf OR leaves OR cuttings OR (Pre-sow* AND (exposure or treatment)) or Flora OR crop* OR plant*) |
