# Supplementary material for: What evidence exists of crop plants response to exposure to static magnetic and electromagnetic fields? A systematic map protocol
Source: Environ Evid. 2022 Dec 6;11:37. doi: 10.1186/s13750-022-00292-w (PMC11378831; doi:10.1186/s13750-022-00292-w)
Supplement: Supplementary file 3 — Additional file 3. Benchmark articles and test of search comprehensiveness. [file 13750_2022_292_MOESM3_ESM.docx]

**Benchmark Articles and Test of Search Comprehensiveness**

| **#** | **Title** | **Reference** | **Retrieved with pilot search string in Web of Science on 18.09.2022 (Yes/ No)** |
| --- | --- | --- | --- |
|  | Treatment of Common Sunflower (Helianthus annus L.) Seeds with Radio-frequency Electromagnetic Field and Cold Plasma Induces Changes in Seed Phytohormone Balance, Seedling Development and Leaf Protein Expression. | Mildažienė V, Aleknavičiūtė V, Žūkienė R, Paužaitė G, Naučienė Z, Filatova I, et al. Treatment of Common Sunflower (Helianthus annus L.) Seeds with Radio-frequency Electromagnetic Field and Cold Plasma Induces Changes in Seed Phytohormone Balance, Seedling Development and Leaf Protein Expression. Sci Rep. 2019;9(1):6437. <https://doi.org/10.1038/s41598-019-42893-5> | Yes |
|  | Intercellular communication in plants: Evidence for two rapidly transmitted systemic signals generated in response to electromagnetic field stimulation in tomato. | Beaubois É, Girard S, Lallechere S, Davies E, Paladian F, Bonnet P, et al. Intercellular communication in plants: evidence for two rapidly transmitted systemic signals generated in response to electromagnetic field stimulation in tomato. Plant Cell Environ. 2007;30(7):834–844.  <https://doi.org/10.1111/j.1365-3040.2007.01669.x> | Yes |
|  | Exposure of maize seeds to stationary magnetic fields: Effects on germination and early growth. | Flórez M, Carbonell MV, Martínez E. Exposure of maize seeds to stationary magnetic fields: Effects on germination and early growth. Environ Exp Bot. 2007;59(1):68–75.  <https://doi.org/10.1016/j.envexpbot.2005.10.006> | Yes |
|  | Effect of Electromagnetic Stimulation on Selected Fabaceae Plants. | Sujak A, Dziwulska-Hunek A, Reszczyńska, E. Effect of Electromagnetic Stimulation on Selected *Fabaceae* Plants. Polish J Environ Stud. 2013;22(3): 893–898. | Yes |
|  | Effect of Pre-Sowing Magnetic Field Treatment on Enzymes and Phytohormones in Pea (Pisum sativum L.) Seeds and Seedlings. | Podleśny J, Podleśna A, Gładyszewska B, Bojarszczuk J. Effect of Pre-Sowing Magnetic Field Treatment on Enzymes and Phytohormones in Pea (Pisum sativum L.) Seeds and Seedlings. Agronomy. 2021;11(3):494. <https://doi.org/10.3390/agronomy11030494> | Yes |
|  | The effects of the electromagnetic fields on the biochemical components, enzymatic and non-enzymatic antioxidant systems of tea Camellia sinensis L. | Azizi SMY, Hosseini Sarghein S, Majd A, Peyvandi M. The effects of the electromagnetic fields on the biochemical components, enzymatic and non-enzymatic antioxidant systems of tea Camellia sinensis L. Physiol Mol Biol Plants. 2019;25(6):1445–1456.  <https://doi.org/10.1007/s12298-019-00702-3> | Yes |
|  | Static magnetic field regulates Arabidopsis root growth via auxin signaling. | Jin Y, Guo W, Hu X, Liu M, Xu X, Hu F, et al. Static magnetic field regulates Arabidopsis root growth via auxin signaling. Sci Rep. 2019;9(1):14384. <https://doi.org/10.1038/s41598-019-50970-y> | Yes |
|  | Extremely low frequency non-uniform magnetic fields induce changes in water relations, photosynthesis and tomato plant growth. | De Souza-Torres A, Sueiro-Pelegrín L, Zambrano-Reyes M, Macías-Socarras I, González-Posada M, García-Fernández D. Extremely low frequency non-uniform magnetic fields induce changes in water relations, photosynthesis and tomato plant growth. Int J Radiat Biol. 2020;96(7):951–957. <https://doi.org/10.1080/09553002.2020.1748912> | Yes |
|  | Growth characteristics of maize seeds exposed to magnetic field. | Vashisth A, Joshi DK. Growth characteristics of maize seeds exposed to magnetic field. Bioelectromagnetics. 2017;38(2):151–157.  <https://doi.org/10.1002/bem.22023> | Yes |
|  | Effect of Pre-sowing Magnetic Field Treatment on Some Biochemical and Physiological Processes in Faba Bean (Vicia faba L. spp. Minor). | Podleśna A, Bojarszczuk J, Podleśny J. Effect of Pre-sowing Magnetic Field Treatment on Some Biochemical and Physiological Processes in Faba Bean (Vicia faba L. spp. Minor). J Plant Growth Regul. 2019;38(3):1153–1160. <https://doi.org/10.1007/s00344-019-09920-1> | Yes |
|  | Arabidopsis cryptochrome is responsive to Radiofrequency (RF) electromagnetic fields. | Albaqami M, Hammad M, Pooam M, Procopio M, Sameti M, Ritz T, et al. Arabidopsis cryptochrome is responsive to Radiofrequency (RF) electromagnetic fields. Sci Rep. 2020;10(1):11260. <https://doi.org/10.1038/s41598-020-67165-5> | Yes |
|  | Magnetic field effects on the magnetic properties, germination, chlorophyll fluorescence, and nutrient content of barley (Hordeum vulgare L.). | Ercan I, Tombuloglu H, Alqahtani N, Alotaibi B, Bamhrez M, Alshumrani R, et al. Magnetic field effects on the magnetic properties, germination, chlorophyll fluorescence, and nutrient content of barley (Hordeum vulgare L.). Plant Physiol Biochem. 2022;170:36–48. <https://doi.org/10.1016/j.plaphy.2021.11.033> | Yes |
|  | Pre-treatment of seeds with static magnetic field improves germination and early growth characteristics under salt stress in maize and soybean. | Kataria S, Baghel L, Guruprasad KN. Pre-treatment of seeds with static magnetic field improves germination and early growth characteristics under salt stress in maize and soybean. Biocatal Agric Biotechnol. 2017;10:83–90.  <https://doi.org/10.1016/j.bcab.2017.02.010> | Yes |
|  | Acceleration of germination and early growth of wheat and bean seedlings grown under various magnetic field and osmotic conditions. | Cakmak T, Dumlupinar R, Erdal S. Acceleration of germination and early growth of wheat and bean seedlings grown under various magnetic field and osmotic conditions. Bioelectromagnetics. 2010;31(2):120–129.  <https://doi.org/10.1002/bem.20537> | Yes |
|  | High frequency (900 MHz) low amplitude (5 V m−1) electromagnetic field: A genuine environmental stimulus that affects transcription, translation, calcium and energy charge in tomato. | Roux D, Vian A, Girard S, Bonnet P, Paladian F, Davies E, et al. High frequency (900 MHz) low amplitude (5 V m−1) electromagnetic field: a genuine environmental stimulus that affects transcription, translation, calcium and energy charge in tomato. Planta. 2008;227(4):883–891.  <https://doi.org/10.1007/s00425-007-0664-2> | Yes |
|  | Short-Term Pre-Germination Exposure to ELF Magnetic Field Does Not Influence Seedling Growth in Durum Wheat (Triticum durum). | Muszyński S, Gagoś M, Pietruszewski S. Short-Term Pre-Germination Exposure to ELF Magnetic Field Does Not Influence Seedling Growth in Durum Wheat (Triticum durum).  Polish J Environ Stud. 2009;18(6):1065–1072. | Yes |
|  | Electromagnetic fields (900 MHz) evoke consistent molecular responses in tomato plants. | Roux D, Vian A, Girard S, Bonnet P, Paladian F, Davies E, et al. Electromagnetic fields (900 MHz) evoke consistent molecular responses in tomato plants. Physiol Plant. 2006;128(2):283–288.  <https://doi.org/10.1111/j.1399-3054.2006.00740.x> | Yes |
|  | Effect of presowing magnetic treatment on properties of pea. | Iqbal M, Haq Z, Jamil Y, Ahmad M. Effect of presowing magnetic treatment on properties of pea. Int. Agrophys. 2012;26(1):25-31. <https://doi.org/10.2478/v10247-012-0004-z> | Yes |
|  | Alleviation of Adverse Effects of Ambient UV Stress on Growth and Some Potential Physiological Attributes in Soybean (Glycine max) by Seed Pre-treatment with Static Magnetic Field. | Kataria S, Baghel L, Guruprasad KN. Alleviation of Adverse Effects of Ambient UV Stress on Growth and Some Potential Physiological Attributes in Soybean (Glycine max) by Seed Pre-treatment with Static Magnetic Field. J Plant Growth Regul. 2017;36(3):550–565.  <https://doi.org/10.1007/s00344-016-9657-3> | Yes |
|  | Pre-sowing static magnetic field treatment for improving water and radiation use efficiency in chickpea (Cicer arietinum L.) under soil moisture stress. | Mridha N, Chattaraj S, Chakraborty D, Anand A, Aggarwal P, Nagarajan S. Pre-sowing static magnetic field treatment for improving water and radiation use efficiency in chickpea (Cicer arietinum L.) under soil moisture stress. Bioelectromagnetics. 2016;37(6):400–408. <https://doi.org/10.1002/bem.21994> | Yes |
|  | The effect of the non-ionizing radiation on cultivated plants of Arabidopsis thaliana (Col.). | Stefi AL, Margaritis LH, Christodoulakis NS. The effect of the non ionizing radiation on cultivated plants of Arabidopsis thaliana (Col.). Flora. 2016;223:114–120.  <https://doi.org/10.1016/j.flora.2016.05.008> | Yes |
|  | Impact of pre-sowing magnetic field exposure of seeds to stationary magnetic field on growth, reactive oxygen species and photosynthesis of maize under field conditions. | Shine MB, Guruprasad KN. Impact of pre-sowing magnetic field exposure of seeds to stationary magnetic field on growth, reactive oxygen species and photosynthesis of maize under field conditions. Acta Physiol Plant. 2012;34(1):255–265. <https://doi.org/10.1007/s11738-011-0824-7> | Yes |
|  | Pre-sowing seed magnetic field treatment influence on germination, seedling growth and enzymatic activities of melon (Cucumis melo L.). | Iqbal M, Haq Z ul, Jamil Y, Nisar J. Pre-sowing seed magnetic field treatment influence on germination, seedling growth and enzymatic activities of melon (Cucumis melo L.). Biocatal Agric Biotechnol. 2016;6:176–183.  <https://doi.org/10.1016/j.bcab.2016.04.001> | Yes |
|  | Pre-sowing magnetic treatments of tomato seeds increase the growth and yield of plants. | De Souza A, Garcí D, Sueiro L, Gilart F, Porras E, Licea L. Pre-sowing magnetic treatments of tomato seeds increase the growth and yield of plants. Bioelectromagnetics. 2006;27(4):247–257.  <https://doi.org/10.1002/bem.20206> | Yes |
