# Supplementary material for: What evidence exists of crop plants response to exposure to static magnetic and electromagnetic fields? A systematic map protocol
Source: Environ Evid. 2022 Dec 6;11:37. doi: 10.1186/s13750-022-00292-w (PMC11378831; doi:10.1186/s13750-022-00292-w)
Supplement: Supplementary file 4 — Additional file 4. Data extraction guide. [file 13750_2022_292_MOESM4_ESM.docx]

Data Extraction Guide

| **Variable** | **Description of variable** | **Definition of variable** |
| --- | --- | --- |
| Reviewer 1: | Primary reviewer who extracts data | Surname and first name |
| Reviewer 2: | Reviewer who cross-checks | Surname and first name |
| Bibliographic information | | |
| Title | Title of the literature |  |
| Authors, Editor, or Organization |  | Surname, first name of author or editor; name of organization |
| Corresponding author | Name and contact of corresponding author | Surname, first name, institutional affiliation, contact |
| Abstract | Abstract of the literature | Text of abstract |
| Keywords | Keywords identifying the study, literature. | Keywords |
| Periodical | Name of the journal, book, proceedings, etc. | Text of the title/ name |
| Year | Year of publishing/ preparing the literature | YYYY |
| Volume | Number of the volume | Number |
| Issue | Issue number | Number |
| Page | Page numbers | Range of numbers |
| DOI/ ISSN/ ISBN |  |  |
| Publication type | Type of Publication | Journal article; book; conference object; report; thesis; technical documentation; other |
| Publication content |  | Primary research; review; meta-analysis; editorial; conference presentation; news report; communication; organizational report |
| Location of study | Country where study was conducted | Coded according to ISO 3166-1 alpha-3 |
| Population parameters | | |
| Name of plant (s) under study | Common name and Latin name |  |
| Classification of plant | Family of plant |  |
| Treated plant (plant organ) | The part of the plant exposed to treatment material | Seeds, pollen, cuttings, whole plant, etc. |
| Plant developmental stage at treatment | Stage of development of the plant during treatment | Pre-cultivation, stage of pre-emergence of seedling, post-emergence growth stage, pre-flowering, post-flowering. |
| Exposure parameters | | |
| Type of treatment material | Static magnetic field or specific electromagnetic radiation | Static magnetic field;  Specific non-ionizing electromagnetic field;  Frequency of non-ionizing electromagnetic field in Hz. |
| Source of treatment | From what material was the static magnetic field or non-ionizing electromagnetic field generated | Description of the set up. |
| Intensity of exposure/ treatment |  | Unit of SMF (in T)  Unit of non-ionizing EMF:   - Electric field strength (in Vm^-1^) of the electric component; - Magnetic field strength (in Am^-1^) of the magnetic component; - Magnetic flux density (in T); - Specific energy absorption rate (Wkg^-1^); - Power density (Wm^-2^) |
| Duration of exposure/ treatment | Length of exposure period of the plant (material) to the SMF or EMF | Seconds (s), minutes (m), hours (h), days of exposure |
| Method and consistency of treatment application | How was the treatment applied? | Intermittent exposure or continuous exposure |
| Comparator parameters | | |
| Type of comparator | Control group with no exposure;  Comparing same plant species under different exposure parameters (source of SMF or EMF, frequency of EMF, duration, method of application);  Same treatment protocols on different plant species;  Different study locations. | May include:   - Treated vs non-treated plant species; - Different plant species; - Different stages of plant development or life cycle; - Different planting materials; - SMF vs EMF treatments; - Different frequencies of non-ionizing EMF; - Different intensity of exposure; - Different duration of exposure; - Intermittent vs continuous exposure; - Laboratory vs greenhouse vs field studies. |
| Outcome parameters | | |
| Level of observed outcome and mechanism of action |  | May include:   - Physiological effects; - Biochemical effects; - Metabolic changes; - Cellular level of action; - Molecular level of action. |
| Growth parameters |  | May include:   - Germination rate; - Emergence rate; - Root growth/ density; - Shoot growth; - Flowering; - Time from planting to yield. |
| Yield parameters |  | May include:   - Root length and weight (fresh/ dry); - Shoot length and weight (fresh/ dry); - Yield of agricultural interest (seeds, oil content, fruits, tubers, leaves). |
| Stress parameters |  | May include:   - Drought/ flooding resistance; - Salt resistance; - Heat resistance; - Cold resistance; - Resistance to pathogens (fungi, bacteria, viruses, nematodes) and pests; - Resistance to pollutants including heavy metals; - Nutrient stress (under/over nutrition); - Resistance to wounding. |
| Study design parameters | | |
| Type of study |  | Experimental study;  Quasi-experimental study. |
| Context/ setting of study | Type of location experiment is conducted | Laboratory, greenhouse, or field. |
| Climate and growth conditions and state of treatment devices |  | May include: climate, temperature, humidity, soil moisture, type of growth medium, pH of growth medium, light conditions, nutrient content, presence of pollutants, and sensitivity of measuring devices. |
